# Identification of biomarker candidates for filarial parasite infections by analysis of extracellular vesicles

**DOI:** 10.3389/fpara.2023.1281092

**Published:** 2023-10-23

**Authors:** Devyn Yates, Lucia S. Di Maggio, Bruce A. Rosa, Robert W. Sprung, Petra Erdmann-Gilmore, R. Reid Townsend, Philip J. Budge, Joseph Kamgno, Makedonka Mitreva, Gary J. Weil, Peter U. Fischer

**Affiliations:** ^1^ Division of Infectious Diseases, Department of Medicine, Washington University School of Medicine, St. Louis, MO, United States; ^2^ Division of Endocrinology, Metabolism and Lipid Research, Department of Medicine, Washington University School of Medicine, St. Louis, MO, United States; ^3^ Department of Cell Biology and Physiology, Washington University School of Medicine, St. Louis, MO, United States; ^4^ Centre for Research on Filariasis and other Tropical Diseases, Yaoundé, Cameroon; ^5^ Faculty of Medicine and Biomedical Sciences, Department of Public Health, University of Yaoundé I, Yaoundé, Cameroon; ^6^ Department of Genetics, Washington University School of Medicine, St. Louis, MO, United States; ^7^ McDonnell Genome Institute, Washington University School of Medicine, St. Louis, MO, United States

**Keywords:** *Brugia malayi*, *Loa loa*, extracellular vesicles, biomarker, filarial parasite

## Abstract

**Background:**

Improved diagnostic tools are needed for detecting active filarial infections in humans. Tests are available that detect adult *W. bancrofti* circulating filarial antigen, but there are no sensitive and specific biomarker tests for brugian filariasis or loiasis. Here we explored whether extracellular vesicles released by filarial parasites contain diagnostic biomarker candidates.

**Methods:**

Vesicles were isolated using VN96-affinity purification from supernatants of short-term *in vitro* cultured *B. malayi* microfilariae (Mf) and analyzed by mass spectrometry (Bruker timsTOF). Parasite-specific proteins were identified by bioinformatic analysis and a protein was called present if supported by ≥ 2 spectra. After validation with cultures parasites, this approach was then used to analyze vesicles isolated from plasma of animals infected with *B. malayi* and from humans with heavy *Loa loa* infections.

**Results:**

Vesicles from Mf cultures contained more than 300 *B. malayi* proteins with high consistency across biological replicates. These included the known Mf excretory antigen BmR1 (AF225296). Over 150 *B. malayi* proteins were detected in vesicles isolated from plasma samples from two infected animals. Vesicles isolated from plasma from 10 persons with high *L. loa* Mf densities contained consistently 21 proteins, 9 of them were supported by at least 5 unique peptides and 7 with spectral counts above 10. The protein EN70_10600 (an orthologue of the *B. malayi* antigen BmR1, GenBank AF225296) was detected in all 10 samples with a total count of 91 spectra and a paralogue (EN70_10598) was detected in 6 samples with a total of 44 spectra.

**Discussion:**

Extracellular vesicles released by filarial parasites *in vitro* and *in vivo* contain parasite proteins which can be reliably detected by mass spectrometry. This research provides the foundation to develop antigen detection assays to improve diagnosis of active filarial infections in humans.

## 1 Introduction

Extracellular vesicles (EV) are classified into subcategories based on their size and mode of biogenesis. Modes of biogenesis include shedding of the plasma membrane and derivation from the endocytic pathway (called exosomes). Typically microvesicles are within the range of 100-1,000 nm in size, with apoptotic bodies falling between 50 and 5,000 nm and exosome like bodies being under 150 nm (Eichenberger et al., 2018). Research into EV has increased in recent years, with a growing interest in understanding the role of EV in disease. A more targeted emphasis has been placed on understanding their ability to communicate with other cells and how the contents of the EV cargo could contain potential diagnostic targets (Schorey et al., 2015; Xu et al., 2016; Eichenberger et al., 2018).

Neglected tropical diseases (NTDs) include a number of helminth infection and studies have shown an important role of EV in the parasite-host interactions. Filarial parasites are of particular interest due to their ability to survive within the host for a number of years, even after drug treatment, because the commonly used drugs do not kill the adult worms efficiently. Filarial parasites are nematodes (roundworms) that are transmitted by arthropod vectors. Adult worms produce microfilaria (Mf), which are taken up by blood-sucking dipterans to spread the infection (Cross, 1996). The three primary filarial diseases in humans are lymphatic filariasis (LF), onchocerciasis and loiasis. LF is caused by three closely related filarial species, *Wuchereria bancrofti*, *Brugia malayi* and *Brugia timori*. Onchocerciasis is caused by *Onchocerca volvulus* and loiasis is caused by *Loa loa*. Among these, only zoophilic strains of *B. malayi* can be maintained in laboratory animals and represent an excellent animal model for other filarial species that infect humans. Diagnostic tests to detect adult *W. bancrofti* circulating antigen are available, but there are no sensitive and specific biomarker tests for brugian filariasis and loiasis, nor biomarkers specifically associated with Mf. This is especially important when working in *L. loa* co-endemic areas, as serious adverse effects are possible after mass drug administration with ivermectin to eliminate LF or onchocerciasis.

Previous studies have shown EV are released by parasitic helminths and may influence tissue repair, transfer of pathogenic proteins and modifying the host immune response (Marcilla et al., 2012). EV make up a large portion of helminth excretory-secretion (ES) products and transport a wide range of proteins within parasitic helminths, confirmed by a variety of methods including proteomics (Marcilla et al., 2012; Jalaludin et al., 2023). ES products of the different filarial life cycle stages, some of which have immunomodulatory properties, have been studied previously as potential biomarkers for infection (Hewitson et al., 2008; Moreno and Geary, 2008; Grainger et al., 2010; Robinson et al., 2013; Drurey et al., 2020). In most studies, ES products have been generated by *in vitro* culture of parasites and analyzed by various methods, including proteomics (Hewitson et al., 2008; Moreno and Geary, 2008; Bennuru et al., 2009; Hewitson et al., 2014). In an artificial culture system it is difficult to differentiate “true ES products” from proteins that are only occasionally set free by stressed, moribund, dead or disintegrating worms. Like other helminths, filarial parasites have been shown to release EV that contain ES products (Zamanian et al., 2015; Harischandra et al., 2018; Loghry et al., 2020; Loghry et al., 2022a), which may be more likely to contain exclusively “true ES products”.

There is a relatively large body of knowledge available about the makeup of EV from *B. malayi.* EV have been shown to be released by *B. malayi* throughout its life cycle, from L3 into adulthood. This was further explored through the use of nanoparticle tracking analysis (NTA) and electron microscopy, which both shown a large size distribution of EV released. Additional analysis used proteomics to study the proteins within these EV and within the miRNA (Zamanian et al., 2015; Harischandra et al., 2018). Other studies looked into how *B. malayi* EV interact with its host, which give new insight into host-parasite relationships (Ricciardi et al., 2021; Loghry et al., 2022a). While there are a number of different protocols to isolate EV for proteomic analysis published (Janouskova et al., 2022; Shirejini and Inci, 2022; Jalaludin et al., 2023), the efficiency and reproducibility of different methods for the isolation of EV produced by filarial parasites has not been compared. The purpose of the present study was to establish a robust protocol to isolate EV from filarial parasites and to explore potential value of filarial proteins present in EV produced *in vitro* or *in vivo* as novel biomarkers for detection of active filarial infection.

## 2 Results/discussion

### 2.1 Evaluation of EV isolation kits from *B. malayi* Mf and Adult Worms

**Figure 1:**
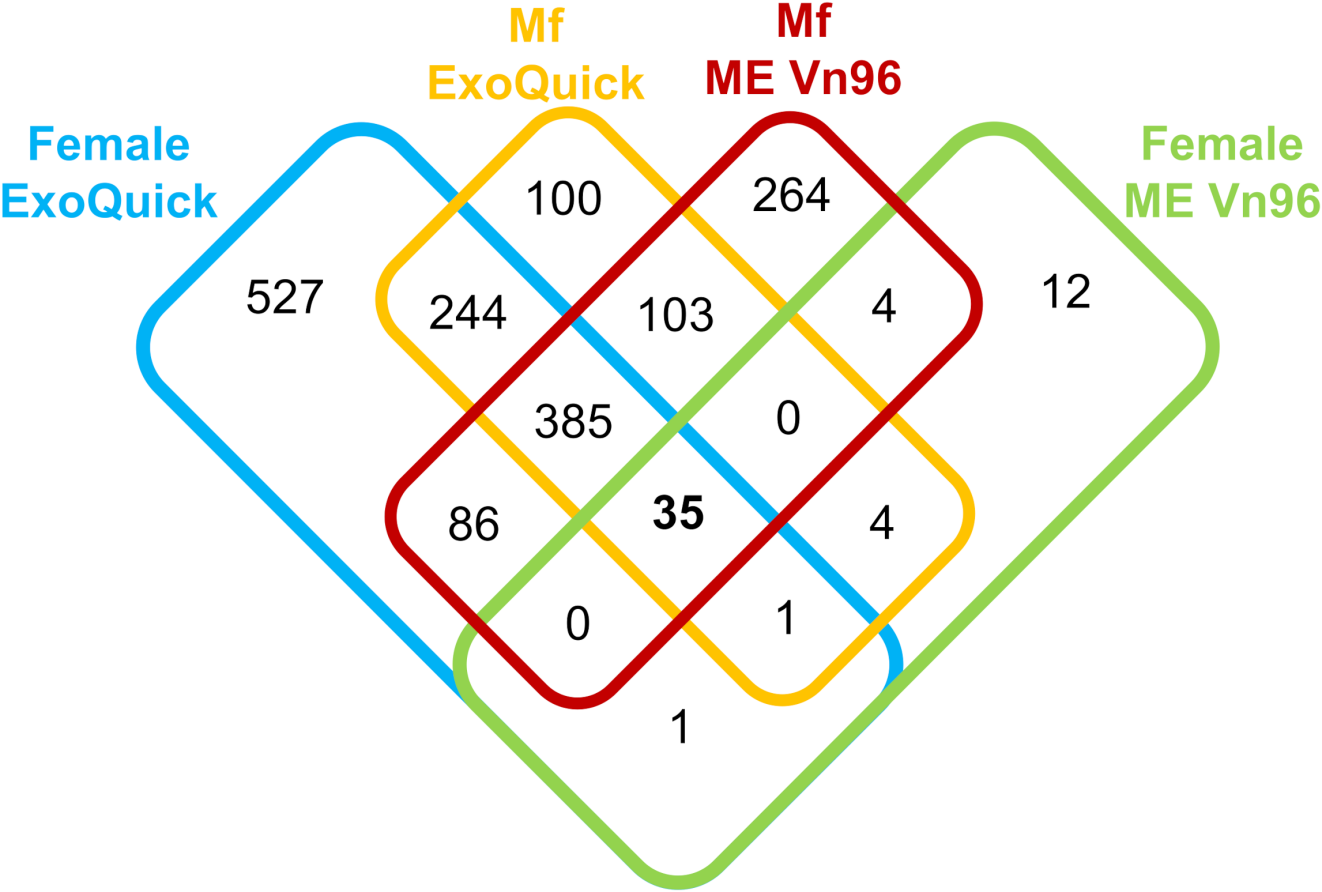
Overview of *Brugia malayi* proteins identified from the ExoQuick TC Ultra kit (left) and the ME Kit for culture media (right) in adult female, and microfilaria samples.

To select the most efficient method for EV isolation, we compared capture of EV from culture supernatants of *B. malayi* adult males, females and Mf with two different kits: ExoQuick TC (SBI) and ME VN96 kit for culture media (Biosynth). A total of 1,766 *B. malayi* proteins were detected by the two kits. Of these, 871 were unique to the ExoQuick kit, 280 unique to the ME kit, and 615 were detected by both kits. Differences were observed in the number of proteins identified in the adult female and Mf samples between the kits (**Figure 1**). The ExoQuick kit found significantly more proteins unique to the adult female sample in comparison to the ME kit, 527 versus 12 respectively. The ME kit identified 264 proteins unique to Mf in comparison to the 100 from the ExoQuick kit. A relatively small number of proteins were detected in the EV of the adult male samples and the numbers were similar for two kits: 14 proteins were identified by the ExoQuick kit and 13 proteins identified by the ME kit. None of these proteins were unique to the adult male, all overlapped with female or Mf samples. Due to this, the male samples were not included in **Figure 1**. Three of these proteins (Bm4277, Bm8524, and Bm4259a) identified in males appeared in all adult female and Mf samples.

Relative protein abundance measured by Normalized Spectral Abundance Factor (NSAF) (Zybailov et al., 2006) values were used to identify the most abundant proteins in the samples. **Table 1** highlights the ten most abundant proteins present in the Mf, adult male and adult female samples in both EV isolation kits. Between the adult male samples, 4 of the 10 most abundant proteins were identified from both kits. For the adult female samples, 3 of the 10 were identified and for the Mf samples 5 of the 10 were identified in both kits. A complete list of all proteins detected with NSAF values is available in **Supplementary Table S2**.

**Table 1:** Top 10 abundant proteins (based on NSAF) present in *B. malayi* adult male, adult female and microfilaria EV isolated from two kits. Proteins are shown in order of highest abundance at the top.

Identified Protein		Description	NSAF	Identified Protein		Description	NSAF
SBI ExoQuick TC				Biosynth Vn96			
Adult Male *B. malayi* Samples							
**Bm8524**	**Actin 2**		0.1535	**Bm13880**	**Triosephosphate isomerase**		0.2824
Bm4112b	Histone H4		0.1121	Bm294a	Bm294		0.1377
Bm4259a	14-3-3-like protein 2		0.1046	**Bm4277**	**Galectin**		0.1090
**Bm4277**	**Galectin**		0.1031	**Bm8524**	**Actin 2**		0.1044
**Bm13880**	**Triosephosphate isomerase**		0.0935	Bm5699	Glyceraldehyde-3-phosphate dehydrogenase		0.0900
Bm4100	Histone H3		0.0849	Bm3146a	Fatty-acid and retinol-binding protein 1		0.0651
Bm3235	BMA-MLC-3		0.0769	Bm6084	uncharacterized		0.0494
**Bm13837**	**ATP synthase subunit alpha**		0.0646	Bm3610	Bm3610		0.0451
Bm13835	ATP synthase subunit beta		0.0421	Bm13965	2-phospho-D-glycerate hydro-lyase		0.0400
Bm5505	Gamma-glutamyltranspeptidase family protein		0.0391	**Bm13837**	**ATP synthase subunit alpha**		0.0325
Adult Female *B. malayi* Samples							
Bm4112b	Histone H4		0.0181	Bm13880	Triosephosphate isomerase		0.1384
Bm3206a	Histone H2B		0.0177	**Bm5699**	**Glyceraldehyde-3-phosphate dehydrogenase**		0.0687
Bm7143	Peptidyl-prolyl cis-trans isomerase		0.0089	**Bm4277**	**Galectin**		0.0572
**Bm4259a**	**14-3-3-like protein 2**		0.0074	Bm294a	Bm294		0.0536
Bm4343a	BMA-PAT-10		0.0069	Bm3146a	Fatty-acid and retinol-binding protein 1		0.0471
**Bm5699**	**Glyceraldehyde-3-phosphate dehydrogenase**		0.0067	Bm7385	Major allergen		0.0453
Bm3210	Small heat shock protein		0.0062	Bm18019	uncharacterized		0.0446
Bm3235	BMA-MLC-3		0.0062	Bm10410	Bm10410		0.0364
Bm4733	Tubulin beta chain		0.0058	Bm5901a	Calmodulin		0.0342
**Bm4277**	**Galectin**		0.0058	**Bm4259a**	**14-3-3-like protein 2**		0.0308
*B. malayi* Microfilaria Samples							
Bm4233b	Venom allergen antigen-like protein 1		0.0301	**Bm4112b**	**Histone H4**		0.0347
**Bm8524**	**Actin 2**		0.0217	**Bm10631**	**Recombinant antigen R1**		0.0231
**Bm3206a**	**Histone H2B**		0.0174	**Bm8524**	**Actin 2**		0.0190
**Bm10631**	**Recombinant antigen R1**		0.0172	**Bm3206a**	**Histone H2B**		0.0167
**Bm4112b**	**Histone H4**		0.0147	**Bm9336a**	**Chitin-binding type-2 domain-containing protein**		0.0145
Bm18051	Serpin Bm1988		0.0138	Bm4259a	14-3-3-like protein 2		0.0135
Bm4277	Galectin		0.0129	Bm2001	DUF148 domain-containing protein		0.0129
Bm8172b	Bm8172		0.0119	Bm4100	Histone H3		0.0118
Bm12956	Bm12956		0.0082	Bm5901a	Calmodulin		0.0092
**Bm9336a**	**Chitin-binding type-2 domain-containing protein**		0.0073	Bm3226	Thioredoxin domain-containing protein		0.0091

Bolded proteins identify those that appear in samples in both kits.

While these pilot studies identified similar numbers of Mf proteins from both kits, we found the ME VN96 kit more easily compatible with sample preparation for LC-MS/MS, and used the ME VN96 kit for all future experiments.

### 2.2 Consistency of ME EV isolation kit with biological replicates

**Figure 2:**
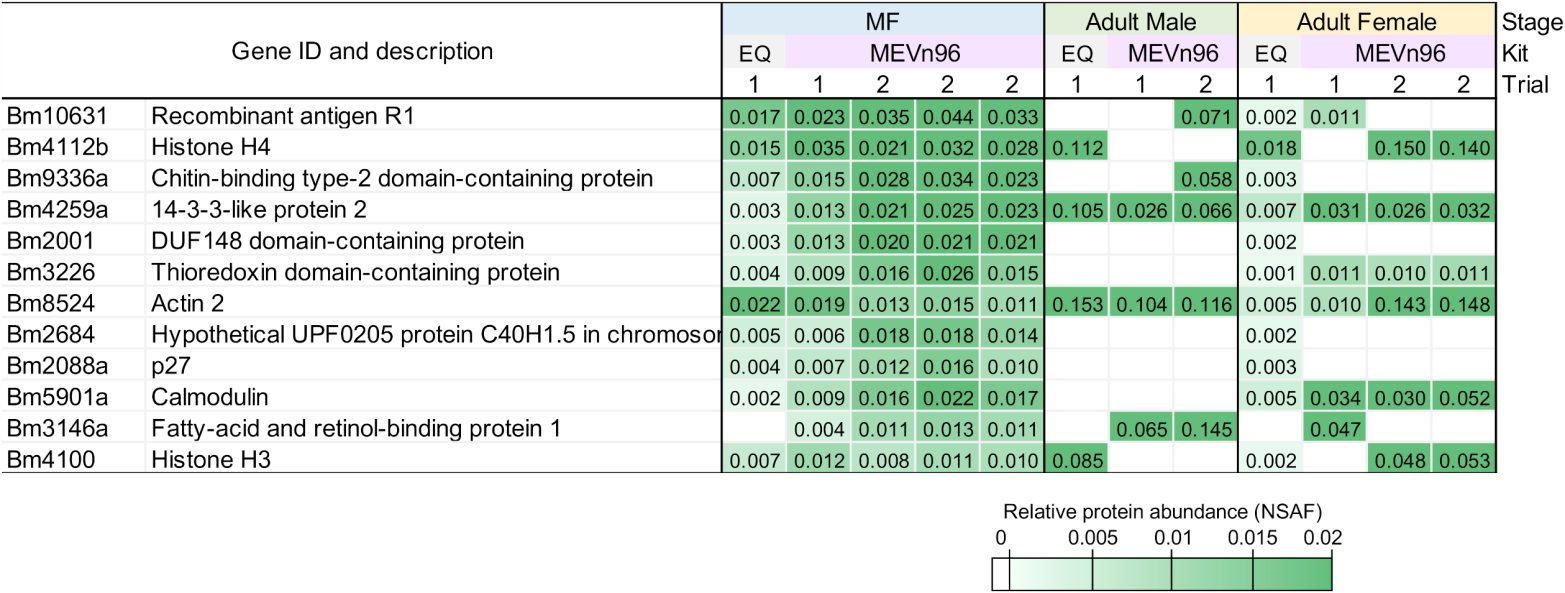
Heat map of the 12 most abundant proteins found in the *B. malayi* Mf samples determined by NSAF. Values are shown for all stages, kits and trials.

To evaluate the consistency of the ME kit, EV were isolated from three separately cultured *B. malayi* Mf samples. In these biological replicates, the total number of proteins identified were 511, 321, and 522. Ten of the top 15 most abundant proteins identified in each sample were detected were in all three biological replicates (**Figure 2**). One of the most abundant was Bm10631, which is identical to BmR1 (GenBank AF225296), a previously characterized *B. malayi* biomarker (Rahmah et al., 2001). We did additional work to confirm that the identification of Bm10631 was supported with multiple peptides over the entirety of the protein (**Supplementary Figure S1**). See **Supplementary Table S3** for the full list of identified proteins and NSAF values.

**Figure 3:**
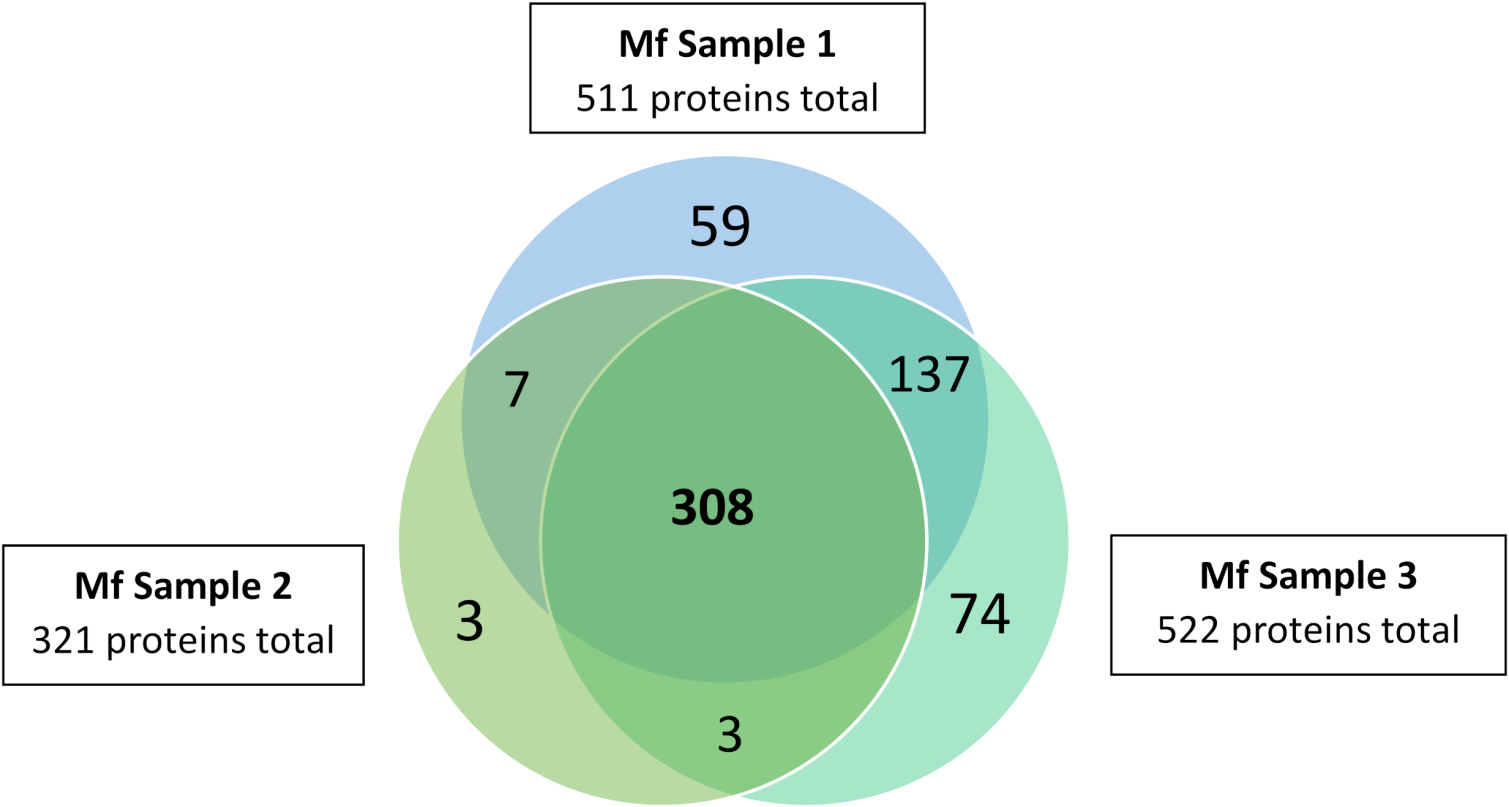
Distribution of proteins identified in three *B. malayi* Mf samples. EV were isolated using the ME kit for culture media and processed using mass spectrometry. 308 proteins were detected in all three replicates and 455 proteins in at least 2 biological replicates.

**Figure 4:**
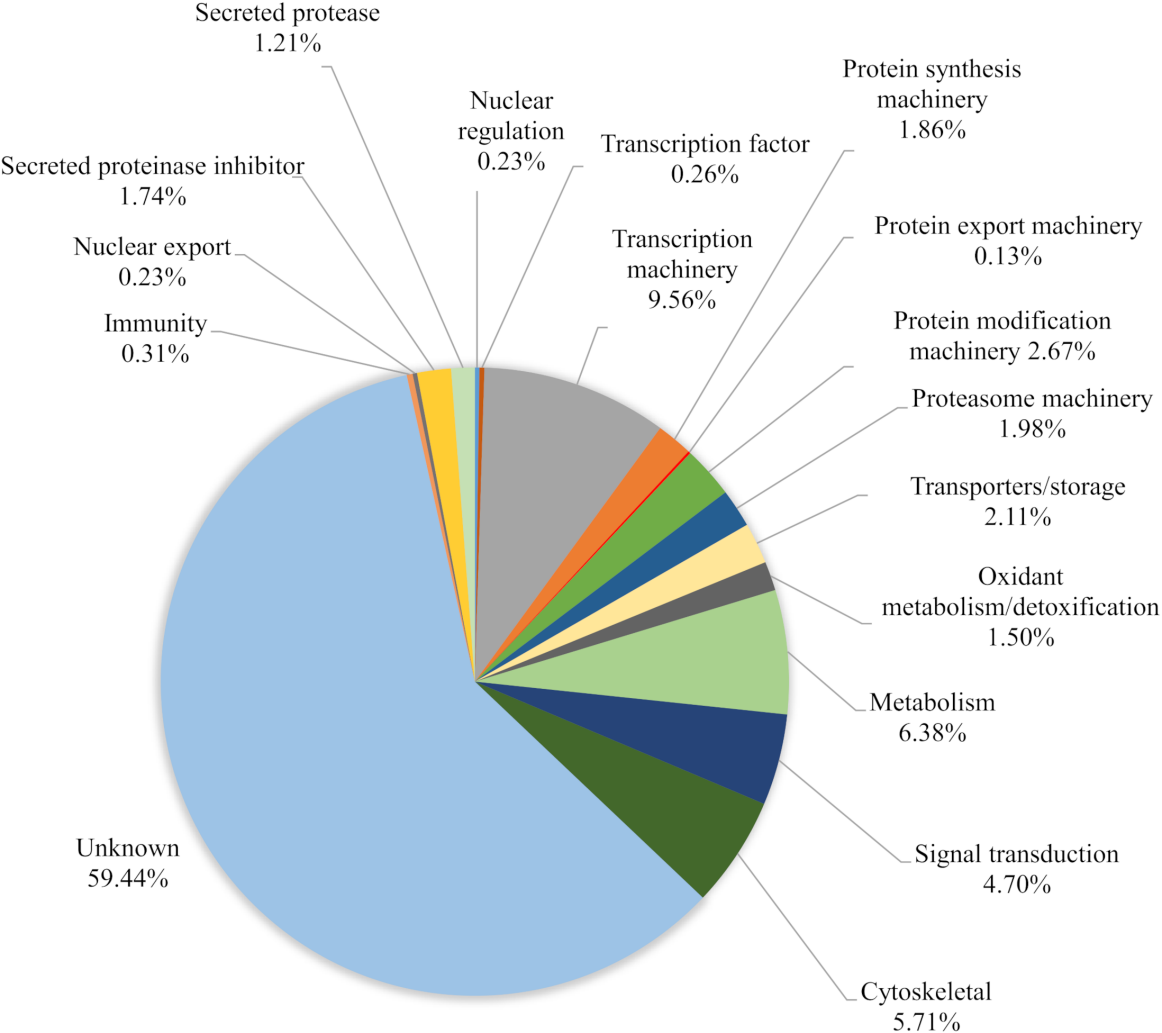
Characterization of the total proteins (591) based on relative abundance calculated from three biological replicates of *B. malayi* Mf. Proteins were identified from EV isolated with the ME kit.

Overall, the ME kit exhibited high consistency from different biological samples within the Mf short-term cultures. A large majority of proteins, 308, were identified in all three biological samples (**Figure 3**). A further 137 proteins were common to the two samples Mf 1 and Mf 3, which had a higher number of total proteins detected than sample Mf 2. Nearly 60% of the proteins found were of unknown or uncharacterized function; among proteins with predicted cellular functions, metabolism, transcription, cytoskeletal synthesis, and signal transduction were heavily represented (**Figure 4**). From the results outlined in **Figures 2** and **3**, we determined that robust reproducibility of results could be achieved in biological samples with the ME kit for culture media. This gave us confidence to begin testing more difficult and diverse samples using plasma collected from hosts infected with *B. malayi*.

### 2.3 Evaluation of ME kit with plasma of animals infected with *B. malayi*

We next isolated EV from the plasma of an infected gerbil. Proteomics analysis identified 110 proteins unique to *B. malayi*. The top 10 hits (based on unique peptide count) are shown in **Table 2**; **Supplementary Table S4** shows the full list of proteins detected.

**Table 2:** Top 10 identified *B. malayi* proteins based on unique peptide count.

Protein	Description	Unique Peptide Count	Total Spectral Count
Bm9228	Tubulin alpha chain	11	59
Bm10379	Tubulin alpha chain	10	55
Bm13733	BMA-EEF-2	9	17
Bm13835	ATP synthase subunit beta	8	14
Bm13916	BMA-PRP-8	8	12
Bm5901	Calmodulin	8	28
Bm6157	Serine/threonine-protein phosphatase	8	16
Bm5003	Calcium-transporting ATPase	7	14
Bm12766	Tubulin beta chain	6	71

Proteins were identified from EV isolated from plasma of infected gerbils.

To validate these results, we next analyzed EV isolated from four plasma samples of an infected cat. To test the sensitivity of our detection, each sample was concentrated to 1mL from a different starting volume (1.5 mL, 2.0 mL, and 4.0 mL) as well as having a 1mL control. A total of 55 proteins and 82 peptides unique to *B. malayi* were detected. Within the four samples, there was little difference noted between the samples in terms of the parasite hits (**Table 3**). The 1mL control plasma sample had 34 proteins identified while the 4mL concentrated sample had 29. The 1.5mL and 2.5mL concentrated samples had 30 and 26 proteins identified respectively. There were 6 proteins (Bm9583, Bm4529, Bm7563, Bm2987, Bm3340, and Bm4484) detected in all samples and technical triplicates. A complete list of unique peptides and corresponding spectra can be found in **Supplementary Table S5**.

**Table 3:** Top 10 proteins identified from plasma of a cat infected with *B. malayi*.

Protein	Description	Total Unique Peptide Counts				Total Spectra Counts			
		1mL	1.5mL	2.5mL	4mL	1mL	1.5mL	2.5mL	4mL
Bm9583	14-3-3 like protein	2	2	2	2	27	31	27	28
Bm4529	Protein kinase C	1	1	1	1	6	11	15	11
Bm2987	BMA-MLC-4	1	1	1	1	10	8	6	10
Bm3340	Guanine nucleotide-binding protein beta subunit 1	1	1	1	2	7	6	8	10
Bm7212	Bma-cand-1	1	1	1	1	17	8	5	10
Bm6285	Uncharacterized	2	2	2	2	4	5	18	8
Bm6157	Serine/threonine-protein phosphatase	1	1	1	1	4	5	6	7
Bm3590	Pyruvate kinase	1	1	1	1	8	10	3	6
Bm7563	CULLIN_2 domain protein	1	1	1	1	6	6	7	6
Bm13653	BMA-DAF-21	1	0	1	1	1	0	1	5

Proteins are sorted by the highest spectral counts of the 4mL sample.

Comparing the cat and gerbil plasma samples, 56 proteins were detected in plasma samples of both species. These proteins included all *B. malayi* proteins, even those with homology to other host and parasite species, since specific peptide detections were required to match *B. malayi*-specific sequences. The probability of 56 proteins being identified by random chance from both the gerbil (n = 110 proteins) and the cat (n = 136 proteins) samples is P < 10^-20^, according to a negative binomial distribution test. Gene ontology (GO) analysis revealed significant enrichment (FDR-adjusted P values ≤ 0.05) for molecular functions including hydrolase activity or binding activity (ribonucleotides, nucleotides, ions, etc.) (**Supplementary Table S5**).

### 2.4 Evaluation of ME kit with plasma of humans with filarial infections

#### 2.4.1 Evaluation of ME kit with plasma of Mf positive humans with *B. timori* infections

We next tested whether EV from naturally infected humans would yield detectable *Brugia* biomarkers. As no suitable plasma samples from subjects with *B. malayi* infection were available, we used samples from individuals infected with the closely related species *B. timori.* We identified 15 proteins unique to *B. timori* in two available human samples. However, the peptide and spectral counts were very low, all under 4. There was minimal overlap between the two samples, with only 2 proteins found in both (**Supplementary Table S6**). One detected protein, the *B. timori* BTMF00016632 is an orthologue to the *B.malayi* serine/threonine protein phosphatase Bm6257 (100% identical amino acid sequences) which was detected in plasma of infected animals. Our detection of few proteins in this sample may be due to the relatively low Mf counts (74 and 134 Mf/mL) and age of the samples.

#### 2.4.2 Evaluation of ME kit with plasma of humans with high *L. loa* Mf load

**Figure 5:**
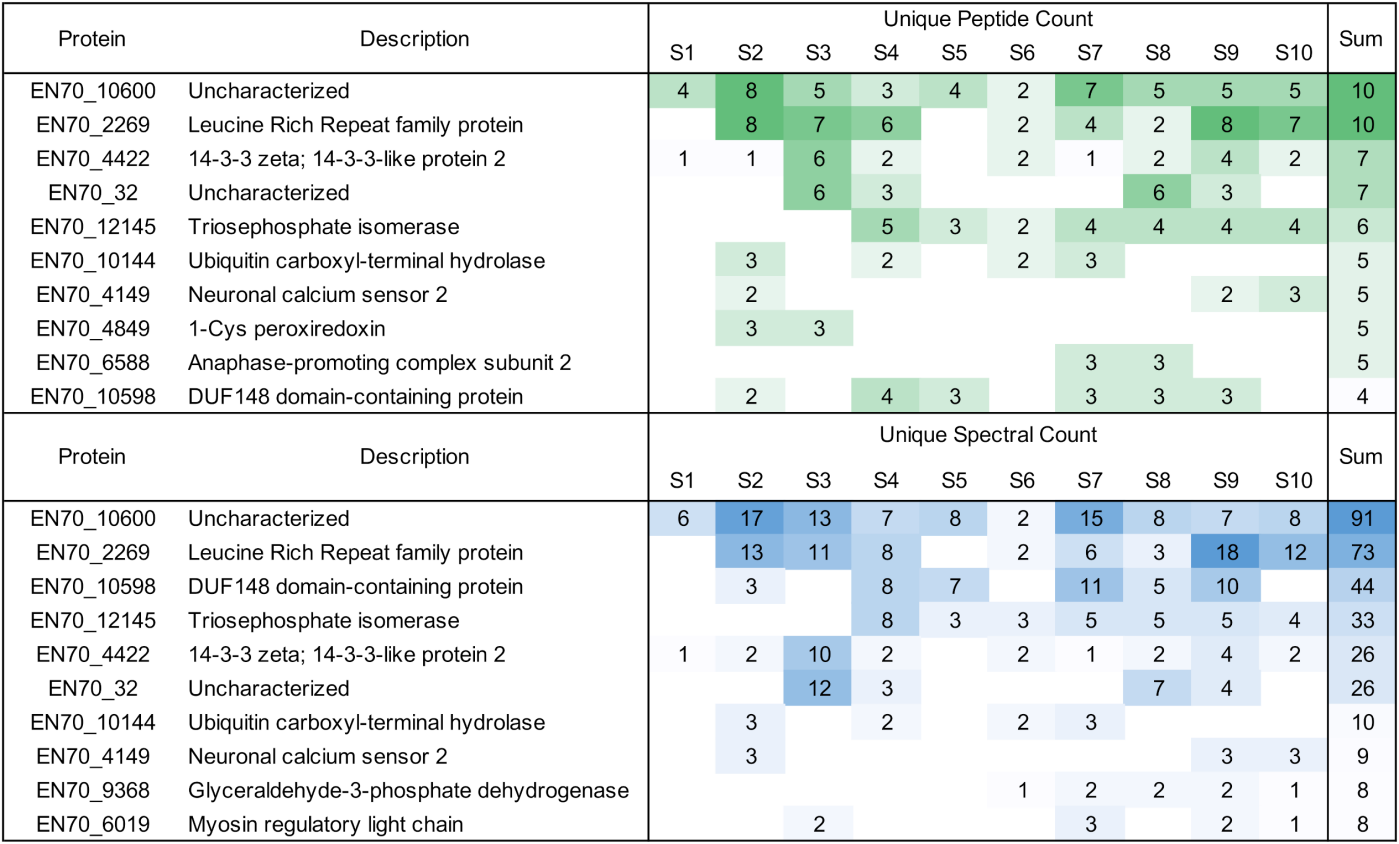
Overview of unique *Loa loa* peptides and spectral counts detected in infected human plasma. Proteins shown are the 10 highest proteins identified by peptide and spectral counts in descending order.

To determine whether EV-borne protein biomarkers would be more easily detected in plasma of individuals with heavy infections, we tested banked plasma samples from 10 individuals with loiasis and high Mf densities (between 29,120 and 81,120 Mf/ml). EV preparations of these samples yielded 21 *L. loa* proteins detected in the 10 samples. **Figure 5** highlights the 10 proteins with the highest unique peptide and spectral counts. The full data set can be found in **Supplementary Table S7**.

The protein EN70_10600 is particularly interesting as it was in all 10 samples with 10 unique peptides and a spectral count of 91. This protein has been shown to have some homology with the Brugia Rapid Test antigen, BmR1 or Bm10631. As in 2.2, we wanted to ensure that we weren’t identifying the same or overlapping peptides in only one portion of EN70_10600. Our alignment shows we had identified peptides throughout the protein (**Supplementary Figure S1**). The protein is a paralogue to EN70_10598 that was detected in 6 samples with a total of 44 spectra.

### 2.5 Discussion

The presented results have shown that the EV released by filarial parasites are a rich source of ES products and characterizing their cargo could lead to identification of biomarkers of infection. Isolation of EV may enrich for filarial proteins compared to total plasma protein that is usually dominated by a relatively small number of highly abundant host proteins. Increasing research with EV released by parasites led to more insights into diseases, such as LF or loiasis, and potentially find new biomarkers that can be used for diagnostic testing.

There is unfortunately no “perfect” method for EV isolation, but many methods are available (Serrano-Pertierra et al., 2019; Janouskova et al., 2022; Shirejini and Inci, 2022; Jalaludin et al., 2023). For our studies, we chose the ExoQuick and ME kits based on previous literature (Ricciardi et al., 2021; Aziz et al., 2022) and collaborator experience (Barlin et al., 2023). There was also research to show that the ME kit was successful for proteomic downstream processing (Ghosh et al., 2014; Knol et al., 2016; Askeland et al., 2020). For EV isolated from cultured *B. malayi* we observed high NSAF values for various histones and cytoskeletal proteins. This has been reported previously by other studies that examined the protein content of vesicles produced by various organisms (Ortiz, 2017; Maan et al., 2018; Ochieng et al., 2018). As shown in Results 2.1, there was variance of the amount of proteins identified in the adult female and Mf samples from the mass spectrometry data. The male worms showed very similar amounts of proteins detected, all of which overlapped with proteins identified in the other types of samples. Other *B. malayi* EV research shows a similar trend of the adult male worm releasing fewer proteins in comparison to the adult female (Harischandra et al., 2018). A previous study on *B. malayi* used differential centrifugation to isolate EV, and we found some proteins in common in the adult male and female samples (Harischandra et al., 2018). These include galectin (Bm4277), 14-3-3 like protein (Bm4259a), and triosephophate isomerase (Bm13880). More recent work shows that galectin contained in EV could play a role in modulating the host immunodulatory response (Loghry et al., 2022b). In another study*, B. malayi* Mf EV were isolated with the ExoQuick kit, which allowed us the most direct comparison of results (Ricciardi et al., 2021). From their list of the 44 proteins identified in all samples (see **Table 1** of (16)), we identified 10 that match directly with our results. Of these, three match to our 25 most abundant proteins identified in the Mf samples isolated from the ExoQuick kit: Bm8524 (actin), Bm5699 (glyceraldehyde-3-phosphate dehydrogenase), and Bm13965 (2-phospho-D-glycerate hydrolyase).

Minimal research was found regarding *B. malayi* EV isolation using the ME kit. For our purposes of a consistent kit for isolating *B. malayi* Mf EV, we completed additional biological replicates. In three biological replicates, we saw over 300 proteins identified in all three samples (over 60%) and over 400 proteins identified in two of the three samples (over 85%). The most abundant protein identified in all three samples was Bm10631, which is identical to BmR1 (GenBank AF225296). The BmR1 antigen is used in the WHO recommended Brugia Rapid test to specifically detect IgG4 antibodies to *Brugia* parasites for monitoring and evaluation of the elimination program (Rahmah et al., 2001). With BmR1 expressed in Mf and adult female worms, it was not surprising to detect this protein in the samples as shown in **Figure 2**. The peptides detected from LC-MS/MS were found across the whole protein (**Supplementary Figure S1**), giving further evidence that Bm10631 was correctly identified in our samples. These results were very promising and encouraged us to move forward with the ME kit in samples more closely resembling clinical Mf counts.

Further experiments were designed with the goal of identifying *B. malayi* EV from infected host plasma. Previous work showed that *B. malayi* immune complexes could be identified from Mf positive sera using LC-MS/MS (Reamtong et al., 2019). A number of proteins identified in this previous work are similar proteins to the ones identified within our experiments, such as heat shock protein (used to isolate the EV), 14-3-3 like protein, and glyceraldehyde 3-phosphate dehydrogenase. A different study found similar proteins in immuno-affinity purified *B. malayi* antigen preparations, specifically in the excretory-secretory products (Hertz et al., 2021). Other studies showed the ability to identify parasite proteins from infected host sera, but these have focused primarily on trematode helminths, and how host serum proteins change after infection (Bi et al., 2021; Guo et al., 2022; Uthailak et al., 2022). There has been limited work studying the isolation of filarial EV from host plasma, but the previous studies serve as a way to somewhat compare results as the *B. malayi* proteins identified in both came from sera.

It was expected that differences would be observed between the EV isolated in culture media and those coming from host sera. In our studies, the gerbil and cat plasma with *B. malayi* infections were much closer to Mf counts sometimes found in humans, although they are still higher than most infections in humans. The GO analysis completed on the 56 proteins in common between the gerbil and cat plasma yielded similar results to previous work looking at the EV isolated from *B. malayi* L3 (Zamanian et al., 2015). Both saw high amounts of proteins associated with metabolic processes such as hydrolases, and binding (ribosomal, nucleotides). This is an interesting finding as different EV isolation methods were completed, different stages of the *B. malayi* life cycle were being studied, and samples came from different origins. It could be hypothesized that many of these proteins are in high abundance due to the function/release of EV.

One unique protein (Bm6157, a serine/threonine-protein phosphatase) was found among the top 10 hits for both the gerbil and cat plasma samples, which was promising. After completing the EV isolation from *B. timori* Mf positive patient sera, we identified the protein BTMF_0001663201, which is a 100% identical ortholog of Bm6157 with a spectra count of 4. While Bm6157 has very high homology amongst the nematode family, the identification of it in infected plasma in two *Brugia* species and three hosts is an important finding. Serine/threonine-protein phosphatase orthologs from other intestinal nematode species have been used as the basis for ruminant vaccines and highlight the similarity between nematodes, including *B. malayi* (Mohamed Fawzi et al., 2013). Another study identified the *O. volvulus* ortholog, OVOC9445 as a top biomarker candidate. It was found in both infected plasma and urine, but only in a single uninfected urine sample supported by a non-unique peptide (Rosa et al., 2023). This gives supportive evidence that EV from plasma can identify similar potential biomarkers across different filarial species.

We moved to *L. loa* patient plasma as these gave us the best chance of detecting Mf EV within the host plasma due to their high Mf densities. There is currently limited information available regarding *L. loa* EV found in patient plasma. Previous studies showed a proteomic analysis of urine samples of a single Mf positive expatriate *L. loa* patient for identifying novel biomarker candidates, which allowed us to have some comparison of results (Drame et al., 2016). The same study used total plasma samples from additional infected subjects to validate four biomarker candidates and two of them continued to show promise after initial validation. We saw no overlap between our identified proteins and their top 4 biomarker candidates. This could be due to a variety of reasons. The previous study focused on only one urine sample, while our study was using 10 sera samples. Our LC-MS/MS was completed with a highly sensitive a timsTOF Pro mass spectrometer, which could contribute to our higher peptide and spectral counts. Our samples also were from individuals in an area endemic for *L. loa*, so their Mf counts were noticeably higher. We did see one protein in both studies: EN70_4422 or LOAG_05701 (14-3-3 like protein), but this protein has high homology with the human ortholog and was not pursued as a biomarker candidate (Drame et al., 2016). The aforementioned study shows the ability to identify viable biomarkers from patient urine using RPLC-MS/MS, showing that our future aims of finding successful biomarkers is possible. Other strategies have been used to identify antigen biomarkers for *L. loa* microfilaremia. One study used transcriptome data for prioritizing candidates and showed some success in detecting antigen biomarker candidates by ELISA with varying degrees of sensitivity and specificity (Drame et al., 2017).

Within the sample set of 10 patient sera, we were able to identify 21 proteins unique to *L. loa*. The number of peptides and spectral counts in these samples shows the possibility of EV to give new biomarker targets to explore. The protein EN70_4422 was identified in 9 of the 10 samples. The homologs for the protein in *B. malayi* are Bm9583, Bm10299 and Bm4259. Of these, we identified Bm4259 in the cultured adult female and Mf samples, as well as in the gerbil plasma. Bm10299 was identified in all four concentrations of the cat plasma samples and Bm9583 was identified in the Mf culture samples. While this protein, 14-3-3 zeta highly conserved in different filarial nematodes, it does support that proteins identified with *L. loa* sera could serve as a way to find biomarkers in other filarial parasites and can give new information regarding host-parasite interactions. This statement is further supported by Results 2.4.2, showing that the protein EN70_10600 was identified in all of the 10 sera samples and EN70_10598 was identified in 6 samples. These two proteins are very similar to one another and are orthologues of BmR1 (Bm10631) (**Supplementary Figure S1**). The identification of a known biomarker (BmR1) and its orthologues (EN70_10600 and EN70_10598) from clinical patient sera gives evidence that EV isolation can lead to discovery of novel biomarkers and increase our understanding of already existing biomarkers in related filarial parasite species.

There were various limitations throughout the different experiments. The biggest limitation overall is the EV isolation methods. With any EV isolation, certain populations will be selected for at higher or lower amounts. Through using a kit that is based on the Vn96 peptide (heat-shock protein), we selected for EV that have this peptide available for binding. While it is very common for this peptide to be associated with EV, we will not know what percentage of EV we are missing using this method. The comparison with the ExoQuick method gives a glimpse into the different proteins that could be identified simply by using a different kit. To better understand the differences between methods and EV populations, more in depth experiments would need to be explored, using differential centrifugation as a standard. Other limitations focus on the lack of variety in samples within trials. This includes that the infected cat plasma came from a single cat. In reference to the *B. timori* plasma samples, these samples were collected in 2002 and the Mf densities were low to moderate (71 and 134 Mf/mL). Additionally, these samples may have gone through a number of freeze thaw cycles which could have reduce the amount of intact EV. All these factors may have played a role in the low peptide and spectral counts of *B. timori* specific peptides identified. Additionally, all *L. loa* originated from patients in Cameroon. Further work could be completed to look at other plasma from infected animals or *L. loa* patient sera from other endemic countries. This would be important to confirming our findings within these experiments.

In summary, we explored new methods for *B. malayi* and *L. loa* EV isolation and analysis for new diagnostic targets through our series of experiments. Our key findings include: [1] the ME kit for EV isolation achieves high consistency in culture media and plasma samples, [2] With mass spectrometry and proteomic analysis it is possible to distinguish parasite EV from host EV in plasma samples. [3] There are major differences between *B. malayi* EV isolated from culture media and those from infected plasma. Future work will focus on expression of some of these identified *L. loa* proteins as well as their homologues other filarial species to develop antigen capture assays.

## 3 Methods

### 3.1 *Brugia malayi* collection and culture

Mongolian gerbils (*Meriones unguiculatus*) were purchased from Charles River Laboratories (Worchester, MA, USA). The 5 to 8 week old male gerbils were kept in an animal care facility at the Department of Comparative Medicine at Washington University in St. Louis (Missouri, USA) under specific pathogen free conditions.

*Brugia malayi* infection: The *B. malayi* life cycle is maintained in *Aedes aegypti* mosquitos (strain “black-eyed Liverpool”) and Mongolian gerbils at an animal care facility at Washington University. Male gerbils were infected by subcutaneous or intraperitoneal injection of 200 L_3_ *B. malayi* (Ash and Riley, 1970). Gerbils were euthanized using a CO_2_ Smart Box (E-Z Systems, Palmer, PA) for single cages. For Methods 1.2 and 1.3, the following protocol was used for media preparation, gerbil dissection and collection of *B. malayi* adult worms and Mf. The worm culture media consisted of RPMI 1640 with 1% glucose and 1% antibiotic antifungal solution (all items from Sigma Aldrich, St. Louis, MO, USA). The media was warmed to 37°C to better reduce shocking the worms post removal from the gerbils. The antibiotic antifungal solution was added right before the worms were collected to ensure as minimal degradation as possible. Gerbils were sacrificed and dissected to collect the parasites from the peritoneal cavity. Adult worms were placed into warmed, filtered PBS (Sigma Aldrich). The cavity was rinsed with filtered PBS and a syringe was used to collect the fluid, containing the majority of the Mf. Adult *B. malayi* worms went through a washing process consisting of three petri dishes containing warm worm culture media. Once the wash process was completed, the adult worms were separated by sex. Mf were collected from the peritoneal cavity PBS rinse and further purified as described below.

### 3.2 EV isolation kit comparison

#### 3.2.1 *In vitro* culture of adults and Mf of *B. malayi*

Following the above process to collect the adult *B. malayi*, culture dishes were prepared with 5mL warm worm culture media. Cultures contained 6 adult female worms in 5 mL and 10 adult male worms in 5 mL, as previously described (Hewitson et al., 2008; Moreno and Geary, 2008; Loghry et al., 2020). To wash and collect the Mf, the samples were spun at 2000 rpm at 10°C for 10 minutes. The Mf pellet was suspended in 5 mL of filtered PBS. The Mf suspension was slowly pipetted into a new conical containing 5 mL of Percoll (Sigma Aldrich). The mixture was centrifuged at 1000 rpm for 10 minutes, followed by a series of centrifugations at 1000rpm to further wash and isolate the Mf. Deionized (DI) water was added to the mixture during one of the steps to ensure that any red blood cells present were disrupted. The Mf pellet was suspended into 30 mL of prepared worm culture media. An estimate was made the following day to collect the density of the Mf using 10 µL of a 1:100 dilution. A total of three slides were averaged to determine the Mf concentration to be approximately 5.1x10^5^ Mf/mL. All adult worms and Mf were placed in an incubator at 37 °C and 5% CO_2_ overnight. After culturing the worms for 24 hours, the worms were removed from the culture media and placed in 100 µL PBS and stored at -80 °C. The media was collected into 15mL conical tubes. Mf samples were collected at ~20 hours of culture. The samples were filtered using a 0.8 µm syringe filter to remove any large debris.

#### 3.2.2 SBI ExoQuick sample preparation

The purification principle of this method is based on the precipitation of EV. Following the ExoQuick TC Ultra (System Biosciences, Palo Alto, CA, USA) protocol, samples were centrifuged at 3000 g for 15 minutes. An optional centrifuge step (10 minutes at 12000g) was completed for the Mf samples to remove any remaining Mf in the sample. All culture media samples (5 mL in volume) had 1 mL of the ExoQuick TC solution (SBI) added to each tube. These incubated overnight at 4 °C. The following day no visible pellet was observed. Samples were centrifuged at 3000 g for 10 minutes. The complete protocol is outlined in **Supplementary Methods S1**.

#### 3.2.3 ME VN96 sample preparation

This method relies on the affinity of EV to the synthetic Vn96 peptide. Following the ME kit for culture media (Biosynth, formerly Vivitide, Gardner, MA, USA) protocol, 4 µL of Vn96 peptide was added to each 1 mL aliquot. Samples rocked at room temperature for 1 hour. Post incubation with the Vn96 peptide, samples were centrifuged to pellet the EV followed by a series of washing and re-pelleting according to the manual. Full details of these steps can be found in **Supplementary Methods S1**. Samples were kept at -80 °C until submission to the mass spectrometry center.

### 3.3 Consistency of ME VN96 EV isolation produced during *in vitro* culture

The methods used for Mf and adult *B. malayi* worms, preparation of the worm culture media, washing of the worms, and suspension of the Mf are the same as described above in Experiment 1.1. The Mf were suspended in 32 mL of culture media at a density of 1.57x10^5^ Mf/mL. All adult worm and Mf samples were cultured at 37 °C and 5% CO_2_ for ~22 hours. Before processing, all culture media samples were filtered using a 0.22 µm filter to remove debris and Mf. A full description of all the protocols completed, including EV isolation can be found in **Supplementary Methods S1**. Samples were kept at -80 °C until submission to the mass spectrometry center.

### 3.4 Isolation of *B. malayi* EV from host plasma

#### 3.4.1 EV isolation from gerbil plasma

About 500 µL venous blood were collected from an adult Mongolian gerbil with a high number of *B. malayi* Mf (20 Mf/10µL). After centrifuging, approximately 300 µl of plasma was collected. An equivalent volume of filtered PBS was added and mixed by inversion. After inversion, the samples were filtered with a 0.22 µm syringe filter to remove Mf from the sample. The filter was flushed with PBS until the final filtered volume was 2 mL. The protocol for EV isolation can be found in **Supplementary Methods S1**.

#### 3.4.2 EV isolation from cat plasma

A whole blood sample of a cat infected with *B. malayi* (approximately 20 mL) was obtained from the NIH NIAID Filariasis Reagent Resource Center (FR3) (Michalski et al., 2011). The infected blood arrived in heparinized tubes at room temperature with an Mf count of 1,400 Mf/mL determined by FR3. Secondary counts were not completed upon arrival of the sample, but samples were reviewed under the microscope to confirm presence of Mf. Centrifugation was used to separate the plasma. To evaluate if the plasma concentration affects the results detected by mass spectrometry, samples were concentrated into 1mL volumes, along with a 1mL un-concentrated sample. The original volumes were 1.5 mL, 2.5 mL and 4 mL of plasma. Concentration was completed using Amicon concentration columns (Millipore Sigma, St. Louis, MO). The plasma was stored at -80 °C for approximately 1 week before EV isolation was completed. Plasma was thawed at 4 °C and stored on ice. The protocol provided by the kit was followed, the full details of this can be found in **Supplementary Methods S1**. The isolated EV were stored at -80 °C until processing.

### 3.5 Isolation filarial EV from human plasma

#### 3.5.1 Isolation *Brugia timori* EV from human plasma

De-identified human plasma samples from individuals who were Mf positive for *B. timori* from Alor, Indonesia, were used for EV isolation. Samples were moved from -80°C storage to -20°C overnight and then on ice to thaw day of processing. Once thawed, PBS was added in equivalent volume to the plasma and inverted to mix. A similar protocol to the previous EV isolations with the VN96 peptide was followed, see **Supplementary Methods S1**. The isolated EV were stored at -80 °C until processing for mass spectrometry.

#### 3.5.2 Isolation *Loa loa* EV from human plasma

EV were isolated form banked, de-identified human plasma samples from individuals in Cameroon with high *L. loa* Mf counts (range 29,000-81,000 Mf/mL). **Supplementary Methods S1** contains a more detailed table of sample information. Samples were moved from -80°C storage to -20°C overnight and then on ice to thaw day of processing. Once thawed, PBS was added in equivalent volume to the plasma and inverted to mix. A similar protocol to the previous EV isolations with the VN96 peptide was followed, see **Supplementary Methods S1**. The isolated EV were stored at -80 °C until processing for mass spectrometry. Three plasma sample from healthy subjects not infected with filarial parasites were included for EV isolation as negative controls, but they did not produce any filarial specific by LC-MS/MS hits and are not mentioned further.

### 3.6 Mass spectrometry

#### 3.6.1 Peptide preparation from solubilized EV pellets

EV pellets were solubilized in 35 µl of SDS buffer (4% (wt/vol), 100 mM Tris-HCl pH 8.0) with sonication in a water bath sonicator (VWR, 150D) at RT for 10min at power level 9. Protein disulfide bonds were reduced using 100 mM dithiothreitol (DTT; Pierce, Waltham, MA) with heating to 95 °C for 10 min. Samples were digested as previously described (Wisniewski et al., 2009). Full details of the digestions and preparation can be found in **Supplementary Methods S1**.

Peptides were purified using 2 different solid phase extraction methods. Purification methods associated with specific samples are annotated in **Supplementary Table S1**. For peptides desalted using Stage tips (Mertins et al., 2018), the peptides were eluted with 60 µl of 50% (vol/vol) acetonitrile (MeCN; J.T. Baker, Radnor Township, PA) in 0.1% (vol/vol) trifluoroacetic acid (TFA; Sigma, St. Louis, MO) and dried in a Speed-Vac (Thermo Scientific, Waltham, MA). For peptides desalted using PGC tips (porous graphite carbon, BIOMETNT3CAR) (Glygen), two micro-tips were used on a Beckman robot (Biomek NXp), as previously described (Chen et al., 2012) for analysis using LC-MS. See **Supplementary Methods S1** for full purification details. The remaining peptides were transferred to autosampler vials (Sun-Sri, Rockwood, TN), dried and stored at -80°C.

#### 3.6.2 Ultra high performance liquid chromatography mass spectrometry - timsTOF

The peptides were analyzed using trapped ion mobility time-of-flight mass spectrometry (Meier et al., 2018). Peptides were separated using a nano-ELUTE chromatograph (Bruker Daltonics. Bremen, Germany) interfaced to a timsTOF Pro mass spectrometer (Bruker Daltonics) with a modified nano-electrospray source (CaptiveSpray, Bruker Daltonics). The mass spectrometer was operated in PASEF mode (Meier et al., 2018). The samples in 2 µl of 1% (vol/vol) FA were injected onto a 75 µm i.d. × 25 cm Aurora Series column with a CSI emitter (Ionopticks, Fitzroy VIC, Australia). The operation conditions can be found in **Supplementary Methods S1**.

#### 3.6.3 Ultra high performance liquid chromatography mass spectrometry – Q-Exactive

The samples were analyzed using ultra-high performance mass spectrometry (Contrepois et al., 2010) on one of three hybrid quadrupole Orbitrap LC-MS/MS systems (Q-Exactive, Thermo Fisher) interfaced to EASY-nano-LC 1000. Instrument-specific parameters are indicated in the machine data files (Proteome Exchange PXD044566). A 75 m i.d. x 50 cm Acclaim PepMap 100 C18 RSLC column (Thermo Scientific, Waltham, MA) was equilibrated with 100% solvent A (1%FA) on the nano-LC for a total of 11 l at 700 bar pressure. Samples in FA 1% (vol/vol) were loaded at a constant pressure of 700 bar. A complete overview of the chromatography is in **Supplementary Methods S1**.

### 3.7 Data analysis

#### 3.7.1 MS data analysis

For timsTOF files, data from the mass spectrometer were converted to peak lists using DataAnalysis (version 5.2, Bruker Daltonics). For Q-Exactive data, unprocessed data from the mass spectrometer were converted to peak lists using Proteome Discoverer (version 2.1.0.81, Thermo-Fischer Scientific). The MS2 spectra from all instruments for peptides with +2, +3 and +4 charge states were analyzed using Mascot software (Perkins et al., 1999) (Matrix Science, London, UK; version 2.5.1). Mascot was set up to search against custom databases corresponding to the parasites and hosts of interest including: *B. malayi* (15,918 entries, PRJNA10729 on WormBase ParaSite (Bolt et al., 2018)), *Meriones unguiculatus* (38,763 entries (Cheng et al., 2019)), *P. kellicotti* (12,850 entries, PRJNA179523 from NCBI (Schoch et al., 2020)), *L. loa* (12,473 entries, PRJNA246086 on WormBase ParaSite (Bolt et al., 2018)), *O. volvulus* (12,224 entries, PRJEB513 on WormBase ParaSite (Bolt et al., 2018)), *W. bancrofti* (13,525 entries, locally improved annotation of GCA_005281725.1 on GenBank (Sayers et al., 2023)) and *Felis catus* (40,213 entries, Uniprot (UniProt, 2023) download August, 2022). Full details of the searches and parameters can be found in **Supplementary Methods S1**. The resulting peptide and protein identifications were imported into Scaffold (Proteome Software, Inc., Portland, OR, USA; v 4.11.1) for visualization and post-processing filtering as described below.

#### 3.7.2 Spectral count analysis

All peptides that were assigned to *B.malayi* and *L. loa* protein databases were searched against protein databases from *M. unguiculatus* and *H. sapiens* using BLASTP, in order to discard any peptides which exactly matched host peptide sequences (considering leucine and isoleucine to be equivalent since they are not distinguished by the MS proteomics approach). After this processing, all peptides and associated spectral counts were parasite-specific sequences. These processed spectral counts and peptide counts for all samples and all detected proteins are provided in **Supplementary Tables S4-S6**. Predicted signal peptides were identified using the SignalP 6.0 server (Teufel et al., 2022) (excluding any proteins with ≥ 2 transmembrane domains, as identified by SignalP). Pathway enrichment was performed using WebGestalt v2019 (Liao et al., 2019) for KEGG pathways (Kanehisa et al., 2021) (Kyoto Encyclopedia of Genes and Genomes) and InterPro domains (Blum et al., 2021) while GOSTATS v2.50 was used for Gene ontology (GO) “molecular function” child term enrichment (Falcon and Gentleman, 2007). InterPro and GO annotations were performed using InterProScan v5.59-91.0 (Jones et al., 2014) and BlastKOALA v2.3 (Kanehisa et al., 2016) was used to annotate KEGG pathways. Relative protein abundance was quantified using Normalized Spectral Abundance Factor (NSAF) values, which are calculated per protein in each sample as the number of spectral counts (SpC) identified for that protein, divided by the protein’s length in amino acids (L), divided by the sum of SpC/L of all protein in the experiment.

## Data availability statement

The original contributions presented in the study are included in the article/supplementary files, further inquiries can be directed to the corresponding author.

## Ethics statement

Ethical approval was not required for the studies involving humans because only de-identified samples were used and this is not considered human subjects research. The studies were conducted in accordance with the local legislation and institutional requirements. The human samples used in this study were acquired from primarily isolated as part of your previous study for which ethical approval was obtained. Written informed consent to participate in this study was not required from the participants or the participants’ legal guardians/next of kin in accordance with the national legislation and the institutional requirements. The animal study was approved by Washington University School of Medicine IACUC. The study was conducted in accordance with the local legislation and institutional requirements.

## Author contributions

DY: Data curation, Formal Analysis, Investigation, Methodology, Visualization, Writing – original draft. LM: Data curation, Formal Analysis, Investigation, Software, Writing – review & editing. BR: Data curation, Formal Analysis, Software, Visualization, Writing – review & editing. RS: Data curation, Formal Analysis, Methodology, Software, Writing – review & editing. PE-G: Formal Analysis, Methodology, Writing – review & editing. RT: Data curation, Resources, Software, Supervision, Writing – review & editing. PB: Resources, Writing – review & editing. JK: Resources, Writing – review & editing. MM: Formal Analysis, Software, Writing – review & editing. GW: Funding acquisition, Project administration, Writing – review & editing. PF: Conceptualization, Funding acquisition, Project administration, Supervision, Writing – review & editing.
