# Supplementary material for: Identification of biomarker candidates for filarial parasite infections by analysis of extracellular vesicles
Source: Front Parasitol. 2023 Oct 23;2:1281092. doi: 10.3389/fpara.2023.1281092 (PMC11732158; doi:10.3389/fpara.2023.1281092)
Supplement: Supplementary file 1 [file DataSheet_1.zip › Supplementary Captions.PDF]

Supplementary Material

1 Supplementary Data

Supplementary Methods S1: This document gives the entirety of the protocols used in each experiment so that others can repeat any methods described.

Supplementary Table S1: Complete list of samples with associated solid phase extraction method, infectious agent, and machine data files. The machine data is available for download at Proteome Exchange: ftp://MSV000092647@massive.ucsd.edu

Supplementary Table S2: Full list of all the NSAF values detected for the vesicle isolation kit comparison with adult male and female worms and microfilaria of *B. malayi*. The NSAF values were imported into Excel using the Scaffold program.

Supplementary Table S3: Full list of all the data used for analysing vesicles isolated from *B. malayi* microfilaria culture with the ME kit. Tab 1 contains the NSAF values. Tab 2 contains the peptide quantitative report. Both of these were imported into Excel using the Scaffold program. Tab 3 contains the data for the GO enrichment.

Supplementary Table S4: Full list of all the peptides detected and identified in *B. malayi* infected plasma from a gerbil. Tab 1 contains all peptides and Tab 2 contains the proteins unique to *B. malayi*. The peptide list was imported into Excel using the Scaffold program.

Supplementary Table S5: Full list of all the peptides detected and identified in *B. malayi* infected plasma from a cat. Tab 1 contains all peptides and Tab 2 contains the proteins unique to *B. malayi*. The peptide list was imported into Excel using the Scaffold program. Tab 3 contains the GO enrichment data for the 56 proteins the infected gerbil and cat plasma had in common.

Supplementary Table S6: Full list of all the peptides detected and identified in *B. timori* infected plasma from a cat. Tab 1 contains all peptides and Tab 2 contains the proteins unique to *B. timori*, along with the peptide and spectral counts. The peptide list was imported into Excel using the Scaffold program.

Supplementary Table S7: Full list of all the peptides detected and identified in *L. loa* infected plasma from a cat. Tab 1 contains all peptides and Tab 2 contains the proteins unique to *L. loa*, along with the peptide and spectral counts. The peptide list was imported into Excel using the Scaffold program.
