# Supplementary material for: Identification of biomarker candidates for filarial parasite infections by analysis of extracellular vesicles
Source: Front Parasitol. 2023 Oct 23;2:1281092. doi: 10.3389/fpara.2023.1281092 (PMC11732158; doi:10.3389/fpara.2023.1281092)
Supplement: Supplementary file 1 [file DataSheet_1.zip › Supplementary Figure 1.PDF]

Supplementary Material

1 Supplementary Figures

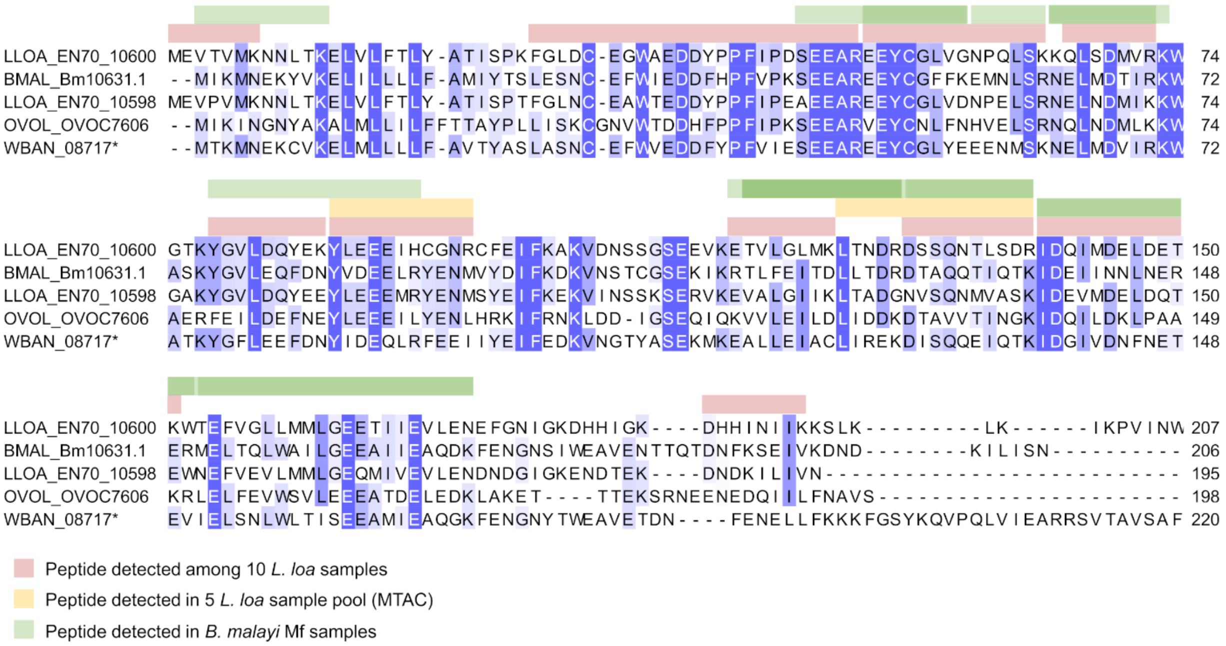

Supplementary Figure 1. Clustal Omega-based protein alignment for *L. loa* EN70_10600, *B. malayi* Bm10631.1, the closely-related EN70_10598, and the top BLAST hits to EN70_10600 in *O. volvulus* (OVOC7606) and *W. bancrofti* (WBAN_08717). Darker blue amino acids indicate more conserved sequences across the species. Shaded areas above the sequences indicate the positions of detected peptides matching to (i) EN70_10600 among the 10 *L. loa* samples (red), (ii) EN70_10600 in the pool of 5 *L. loa* samples (yellow), and (iii) Bm10631 in the *B. malayi* samples (green). Darker shades indicate overlapping peptides in each sample group. *WBAN_08717 continues for another 513 amino acids with no additional alignment from other species.
