# Supplementary material for: Identification of biomarker candidates for filarial parasite infections by analysis of extracellular vesicles
Source: Front Parasitol. 2023 Oct 23;2:1281092. doi: 10.3389/fpara.2023.1281092 (PMC11732158; doi:10.3389/fpara.2023.1281092)
Supplement: Supplementary file 1 [file DataSheet_1.zip › Supplementary Methods.pdf]

Supplementary Methods

3.1 *Brugia malayi* collection and culture

Mongolian gerbils (*Meriones unguiculatus*) were purchased from Charles River Laboratories (Worchester, MA, USA). The 5 to 8 week old male gerbils were kept in an animal care facility at the Department of Comparative Medicine at Washington University in St. Louis (Missouri, USA) under specific pathogen free conditions.

Brugia malayi infection: The *B. malayi* life cycle is maintained in *Aedes aegypti* mosquitos (strain "black-eyed Liverpool") and Mongolian gerbils at an animal care facility at Washington University. Male gerbils were infected by subcutaneous or intraperitoneal injection of 200 L_3 *B. malayi* (Ash and Riley, 1970). Gerbils were euthanized using a CO_2 Smart Box (E-Z Systems, Palmer, PA) for single cages. For Methods 1.2 and 1.3, the following protocol was used for media preparation, gerbil dissection and collection of *B. malayi* adult worms and Mf. The worm culture media consisted of RPMI 1640 with 1% glucose and 1% antibiotic antifungal solution (all items from Sigma Aldrich, St. Louis, MO, USA). The media was warmed to 37°C to better reduce shocking the worms post removal from the gerbils. The antibiotic antifungal solution was added right before the worms were collected to ensure as minimal degradation as possible. Gerbils were sacrificed and dissected to collect the parasites from the peritoneal cavity. Adult worms were placed into warmed, filtered PBS (Sigma Aldrich). The cavity was rinsed with filtered PBS and a syringe was used to collect the fluid, containing the majority of the Mf. Adult *B. malayi* worms went through a washing process consisting of three petri dishes containing warm worm culture media. Once the wash process was completed, the adult worms were separated by sex. Mf were collected from the peritoneal cavity PBS rinse and further purified as described below.

3.2 EV isolation kit comparison

3.2.1 *In vitro* culture of adults and Mf of *B. malayi*

Following the above process to collect the adult *B. malayi*, culture dishes were prepared with 5mL warm worm culture media. Cultures contained 6 adult female worms in 5 mL and 10 adult male worms in 5 mL, as previously described (Hewitson et al., 2008; Moreno and Geary, 2008; Loghry et al., 2020). To wash and collect the Mf, the samples were spun at 2000 rpm at 10℃ for 10 minutes. The Mf pellet was suspended in 5 mL of filtered PBS. The Mf suspension was slowly pipetted into a new conical containing 5 mL of Percoll (Sigma Aldrich). The mixture was centrifuged at 1000 rpm for 10 minutes, followed by a series of centrifugations to further wash and isolate the Mf. Deionized (DI) water was added to the mixture during one of the steps to ensure that any red blood cells present were disrupted. The Mf pellet was suspended into 30 mL of prepared worm culture media. An estimate was made the following day to collect the density of the Mf using 10 µL of a 1:100 dilution. A total of three slides were averaged to determine the Mf concentration to be approximately 5.1×10^5 Mf/mL. All adult worms and Mf were placed in an incubator at 37 ℃ and 5% CO_2 overnight. After culturing the worms for 24 hours, the worms were removed from the culture media and placed in 100 µL PBS and frozen at -80 ℃. The media was collected into 15mL conical tubes. Mf samples were collected at ~20 hours of culture. The samples were filtered using a 0.8 µm syringe filter to remove any large debris.

3.2.2 SBI ExoQuick sample preparation

Following the ExoQuick TC Ultra (System Biosciences, Palo Alto, CA, USA) protocol, samples were centrifuged at 3000 g for 15 minutes. An optional centrifuge step was completed for the Mf samples to remove any remaining Mf in the sample. All culture media samples (5 mL in volume)

had 1 mL of the ExoQuick TC solution (SBI) added to each tube. These incubated overnight at 4 ℃. The following day no visible pellet was observed. Samples were centrifuged at 3000 g for 10 minutes. Centrifugation at 3000g for 10 minutes was completed twice more to remove any remaining ExoQuick solution. Approximately 10 µL of the supernatant was left in each tube and they were frozen at -80 ℃ until further processing. The pellets were not suspended in 200 µL of Buffer B as suggested by the SBI protocol. This was due to not knowing was the contents of Buffer B was but also it was preferred to have the EV in a pellet format for LC/MS. The additional Purification of Isolated EV and Sample Elution protocols in the manual were not completed. This was due to the concern of washing away some of the isolated EV and not knowing what Buffer A and Buffer B were made of, which was a concern for LC/MS analysis.

3.2.3 ME VN96 sample preparation

Following the ME kit for culture media (Biosynth, formerly Vivitide, Gardner, MA, USA) protocol, 4 µL of Vn96 peptide was added to each 1 mL aliquot. Samples rocked at room temperature for 1 hour. Post incubation with the Vn96 peptide, samples were centrifuged at 15,000g for 10 minutes at 4 ℃ to pellet the EV. The pellet was washed with 2 mL PBS followed by centrifugation at 15,000g for 10 minutes at 4 ℃. Washing was completed twice. Samples were kept at -80 ℃ until submission to the mass spectrometry center.

3.3 Consistency of ME VN96 EV isolation produced during *in vitro* culture

The *B. malayi* adult worms and Mf were collected and washed as described above. Worm culture media composition was the same as in experiment 1.1. Large petri dishes were prepared with 20 mL of prepared culture media and warmed to 37 ℃. This larger volume was to ensure that the kit comparison would have the same starting material to remove any variability. The cultures

consisted of 58 adult males in 20 mL and 35 adult females in 20 mL. Additional cultures were prepared with 17 adult females in 8 mL of media. As in Experiment 1.1, the Mf were primarily contained in the PBS that was used to rinse the peritoneal cavity. To isolate and wash the Mf, the samples were spun at 2000 rpm and 10 °C for 10 minutes. The pellets were suspended in a total of 5 mL of PBS and then combined. The suspended pellet was added slowly to 5 mL of HistoPaque (Sigma Aldrich), followed by centrifugation at 1000 rpm for 10 min. Two wash steps were completed using PBS followed by centrifugation at 1000 rpm for 10 min. A total of 3mL DI water was added to the pellet to lyse any red blood cells. After 15 seconds, an additional 12 mL of PBS was added. The previously described wash steps were completed twice more. The Mf were suspended in 32 mL of culture media at a density of $1.57x10^5$ Mf/mL. All adult worm and Mf samples were cultured at 37 °C and 5% CO_2 for ~22 hours. Before processing, all culture media samples were filtered using a 0.22 µm filter to remove debris and Mf.

After filtration with the 0.22 µm filter, samples were centrifuged at 17000 g for 15 minutes. Since each sample had a 5 mL volume, samples were divided into 1 mL aliquots for processing. Following the kit protocol, 4 µL of Vn96 peptide was added to each 1 mL aliquot. For the male and female media, samples rocked at room temperature for 1hr, while the Mf samples rocked overnight at 4°C due to time constraints. Post incubation with the Vn96 peptide, samples were centrifuged to pellet the EV followed by a series of washing and re-pelleting according to the manual (described above in Section 1.2.3). Pellets were combined following the manual as well. Samples were kept at -80 °C until submission to the mass spectrometry center.

3.4 Isolation of *B. malayi* EV from host plasma

3.4.1 EV isolation from gerbil plasma

A small amount of blood was collected from a Mongolian gerbil with a high number of *B. malayi* Mf (20 Mf/10uL). The blood was centrifuged at 800 g for 10 minutes followed by two spins at 2500 g for 20 minutes to separate the plasma. Post centrifugation, approximately 300 µl of plasma remained. An equivalent volume of filtered PBS was added and mixed by inversion. After inversion, the samples were filtered with a 0.22 µm syringe filter to remove Mf from the sample. The filter was flushed with PBS until the final filtered volume was 2 mL. The standard kit protocol was adjusted as the sample volumes were not a full milliliter. To address the lower plasma volume, only 15 µL of the VN96 peptide was added to the sample and then it rotated for 1 hour at room temperature. The recommended centrifugation steps were followed to pellet and wash the isolated EV (described above in Section 1.2.3). The isolated EV were stored at -80 °C until processing.

3.4.2 EV isolation from cat plasma

A whole blood sample of a cat infected with *B. malayi* (approximately 20 mL) was obtained from the Filariasis Reagent Resource Center (FR3) (Michalski et al., 2011). The infected blood arrived in heparinized tubes at room temperature with an Mf count of 1,400 Mf/mL determined by FR3. Secondary counts were not completed upon arrival of the sample, but samples were reviewed under the microscope to confirm presence of Mf. The blood was centrifuged at 800 g for 10 minutes followed by two spins at 2500 g for 20 minutes to separate the plasma. To evaluate if the plasma concentration affects the results detected by mass spectrometry, samples were concentrated into 1mL volumes, along with a 1mL un-concentrated sample. The original volumes were 1.5 mL, 2.5 mL and 4 mL of plasma. Concentration was completed using Amicon concentration columns (Millipore Sigma, St. Louis, MO). The plasma was stored at -80 °C for approximately 1 week before EV isolation was completed. Plasma was thawed at 4 °C and stored

on ice. As per the protocol, 5 µL of ProteaseArrest (G Biosciences, St. Louis, MO) was added to each 1mL sample and 1mL of filtered PBS was added, with inversion of the tubes completed after each addition. Each sample was then filtered using a 0.22 µm syringe filter. To ensure all plasma was pushed through the filter, PBS was added to the syringe to wash the filter out and get the final sample volume to 2 mL. The Vn96 peptide was added to each sample and they rotated for 1 hour at room temperature. The recommended centrifugation steps were followed to pellet and wash the isolated EV (described above in Section 1.2.3). The isolated EV were stored at -80 °C until processing.

3.5 Isolation filarial EV from human plasma

3.5.1 Isolation *Brugia timori* EV from human plasma

De-identified human plasma samples from individuals who were Mf positive for *B. timori* from Alor, Indonesia, were used for EV isolation. Samples were moved from -80°C storage to -20°C overnight and then on ice to thaw day of processing. Once thawed, PBS was added in equivalent volume to the plasma and inverted to mix. Following the protocol, 4 µL of ProteaseArrest was added to the sample and mixed. After inversion, the samples were filtered with a 0.22 µm syringe filter to remove Mf from the sample. The filter was flushed with PBS until the final filtered volume was 2 mL. The standard kit protocol was adjusted as the sample volumes were not a full milliliter. One of the samples had 500 µL plasma and other had 750 µL plasma. To account for this, the volume of VN96 peptide added to the sample was decreased. For the 500 µL sample, 20 µL of peptide was added. The standard 40 µL of peptide was added to the other sample and they were rotated for 1 hour at room temperature. The recommended centrifugation steps were followed to pellet and was the isolated EV (described above in Section 1.2.3). The isolated EV were stored at -80 °C until processing.

3.5.2 Isolation *Loa loa* EV from human plasma

EV were isolated form banked, de-identified human plasma samples from individuals in Cameroon with high *L. loa* Mf counts (range 29,000-81,000 Mf/mL Banked, de-identified human plasma samples from individuals with high *L. loa* Mf counts (Budge et al. Unpublished) were used for the EV isolation. **Table 1** gives further information regarding the samples that were used for this experiment.

Table 1: Overview of *Loa loa* sample data for Experiment 1.5. All villages were within the Okola District in Cameroon. TBF signifies the total number of Mf in each sample determined by a thick blood smear.

Sample	Screen Date	Sex	Age	Day TBF II Mf/ml
1	30/01/2020	F	38	29,960
2	24/01/2020	M	33	50,960
3	31/01/2020	M	29	33,580
4	26/01/2020	F	76	32,060
5	24/01/2020	F	67	42,020
6	25/01/2020	F	61	43,060
7	03/02/2020	M	38	81,120
8	05/02/2020	F	54	48,100
9	06/02/2020	F	46	49,440
10	07/02/2020	M	59	29,120

Samples were moved from -80°C storage to -20°C overnight and then on ice to thaw day of processing. Once thawed, PBS was added in equivalent volume to the plasma and inverted to mix. Following the protocol, 4 µL of ProteaseArrest was added to the sample and mixed. After inversion, the samples were filtered with a 0.22 µm syringe filter to remove Mf from the sample. The filter was flushed with PBS until the final filtered volume was 2 mL. The standard kit

protocol was adjusted as the sample volumes were not a full milliliter. Two of the samples had 500 µL plasma and the remaining 8 had 750 µL plasma. To account for this, the volume of VN96 peptide added to the sample was decreased. For the 500 µL samples, 20 µL of peptide was added. The standard 40 µL of peptide was added to the remaining samples and they were rotated for 1 hour at room temperature. The recommended centrifugation steps were followed to pellet and was the isolated EV (described above in Section 1.2.3). The isolated EV were stored at -80 °C until processing. Three plasma sample from healthy subjects not infected with filarial parasites were included for EV isolation as negative controls, but they did not produce any filarial specific by LC-MS/MS hits and are not mentioned further.

3.6: Mass spectrometry

3.6.1 Peptide preparation from solubilized exosome pellets

EV pellets were solubilized in 35 µl of SDS buffer (4% (wt/vol), 100 mM Tris-HCl pH 8.0) with sonication in a water bath sonicator (VWR, 150D) at RT for 10min at power level 9. Protein disulfide bonds were reduced using 100 mM dithiothreitol (DTT; Pierce, Waltham, MA) with heating to 95 ºC for 10 min. Samples were digested as previously described (Wisniewski et al., 2009). Reduced samples were mixed with 200 µL 100 mM Tris-HCL buffer, pH 8.5 containing 8 M urea (Sigma Aldrich) (UA buffer) and transferred to the top of a 30,000 molecular weight cut-off filter (Millipore Sigma) and spun in a microcentrifuge (Eppendorf, Hamburg, Germany) at 10,000 rcf for 10 minutes. An additional 300 µL of UA buffer was added and the filter was spun at 10,000 rcf for 10 minutes in a microcentrifuge. The flow through was discarded and the proteins were alkylated using 100 µL of 50 mM Iodoacetamide (IAM, Pierce, Waltham, MA) in UA buffer. IAM in UA buffer was added to the top chamber of the filtration unit. The samples were gyrated at 550 rpm using a thermomixer (Eppendorf) at room temperature for 30 minutes in

the dark. The filter was spun at 10,000 rcf for 10 minutes and the flow through discarded. Unreacted IAM was washed through the filter with two additions of 200 µL of UA buffer, and centrifugation at 10,000 rcf for 10 minutes after each buffer addition. The UA buffer was exchanged with digestion buffer (DB), 50 mM ammonium bicarbonate buffer, pH 8. Two sequential additions of DB (200 µL) with centrifugation after each addition to the top chamber was performed. The filters were transferred to a new collection tube, then trypsin (Promega, Madison, WI) (1:20) in DB buffer was added to the top of the filter and samples were digested overnight at 37 °C. The filters were spun at 14,000 rcf for 15 minutes to collect the peptides in the flow through. The filter was washed with 50 µL 100 mM ammonium bicarbonate buffer and the wash was collected with the peptides. In preparation for desalting, peptides were acidified to 1% (vol/vol) TFA final concentration.

Peptides were purified using 2 different solid phase extraction methods. Purification methods associated with specific samples are annotated in **Supplementary Table 1**. For peptides desalted using Stage tips (Mertins et al., 2018), the peptides were eluted with 60 µl of 50% (vol/vol) Acetonitrile (MeCN; J.T. Baker, Radnor Township, PA) in 0.1% (vol/vol) Trifluoroacetic acid (TFA; Sigma, St. Louis, MO) and dried in a Speed-Vac (Thermo Scientific, Waltham, MA). Samples were then dissolved in 20 µl of 1% (vol/vol) acetonitrile in water. An aliquot (10%) was removed for quantification using the Pierce Quantitative Fluorometric Peptide Assay kit (Thermo Scientific, Waltham, MA).

For peptides desalted using PGC tips (porous graphite carbon, BIOMETNT3CAR) (Glygen), two micro-tips were used on a Beckman robot (Biomek NXp), as previously described (Chen et al., 2012) for analysis using LC-MS. The peptides were eluted with 60% acetonitrile in 0.1%

TFA and dried in a Speed-Vac (Thermo Scientific, Waltham, MA. The remaining peptides were transferred to autosampler vials (Sun-Sri, Rockwood, TN), dried and stored at -80 °C.

3.6.2 Ultra high performance liquid chromatography mass spectrometry - timsTOF

The peptides were analyzed using trapped ion mobility time-of-flight mass spectrometry (Meier et al., 2018). Peptides were separated using a nano-ELUTE chromatograph (Bruker Daltonics. Bremen, Germany) interfaced to a timsTOF Pro mass spectrometer (Bruker Daltonics) with a modified nano-electrospray source (CaptiveSpray, Bruker Daltonics). The mass spectrometer was operated in PASEF mode (Meier et al., 2018). The samples in 2 µl of 1% (vol/vol) FA were injected onto a 75 µm i.d. × 25 cm Aurora Series column with a CSI emitter (Ionopticks). The column temperature was set to 50 °C. The column was equilibrated using constant pressure (800 bar) with 8 column volumes of solvent A (0.1% (vol/vol) FA). Sample loading was performed at constant pressure (800 bar) at a volume of 1 sample pick-up volume plus 2 µl. The peptides were eluted using one column separation mode with a flow rate of 300 nL/min and using solvents A (0.1% (vol/vol) FA) and B (0.1% (vol/vol) FA/MeCN): solvent A containing 2% B increased to 17% B over 60 min, to 25% B over 30 min, to 37% B over 10 min, to 80% B over 10 min and constant 80% B for 10 min. The MS1 and MS2 spectra were recorded from m/z 100 to 1700.

The collision energy was ramped stepwise as a function of increasing ion mobility: 52 eV for 0–19% of the ramp time; 47 eV from 19–38%; 42 eV from 38–57%; 37 eV from 57–76%; and 32 eV for the remainder. The TIMS elution voltage was calibrated linearly using the Agilent ESI-L Tuning Mix (m/z 622, 922, 1222).

3.6.3 Ultra high performance liquid chromatography mass spectrometry – Q-Exactive.

The samples were analyzed using ultra-high performance mass spectrometry (Contrepois et al., 2010) on one of three hybrid quadrupole Orbitrap LC-MS systems (Q-Exactive, Thermo Fisher) interfaced to EASY-nano-LC 1000. Instrument-specific parameters are indicated in the machine data files (Proteome Exchange PXD044566).

A 75 m i.d. x 50 cm Acclaim PepMap 100 C18 RSLC column (Thermo Scientific, Waltham, MA) was equilibrated with 100% solvent A (1%FA) on the nano-LC for a total of 11 l at 700 bar pressure. Samples in FA 1% (vol/vol) were loaded at a constant pressure of 700 bar. Peptide chromatography was initiated with mobile phase A (1% FA) containing 2% solvent B (100%ACN, 1%FA) for 5 min, then increased to 20% B over 100 min, to 32% B over 20 min, to 95% B over 1 min and held at 95% B for 19 min, with a flow rate of 250 nL/min. Data were acquired in data-dependent mode. Full-scan mass spectra were acquired with the Orbitrap mass analyzer using a scan range of m/z = 325 to 1800 and a mass resolving power set to 70,000. Ten data-dependent high-energy collisional dissociations were performed with a mass resolving power at 17,500, a fixed lower value of m/z 110, an isolation width of 2.0 Da, and a normalized collision energy setting of 27. The maximum injection time was 60 ms for parent-ion analysis and product-ion analysis. Ions that were selected for MS/MS were dynamically excluded for 30 sec. The automatic gain control (AGC) was set at a target value of 1e6 ions for full MS scans and 1e5 ions for MS2.

3.7 Data analysis

3.7.1 MS data analysis: For timsTOF files, data from the mass spectrometer were converted to peak lists using DataAnalysis (version 5.2, Bruker Daltonics). For Q-Exactive data, unprocessed data from the mass spectrometer were converted to peak lists using Proteome Discoverer (version 2.1.0.81, Thermo-Fischer Scientific). The MS2 spectra from all instruments for peptides

with +2, +3 and +4 charge states were analyzed using Mascot software (Perkins et al., 1999) (Matrix Science, London, UK; version 2.5.1). Mascot was set up to search against custom databases corresponding to the parasites and hosts of interest including: *B. malayi* (15,918 entries, PRJNA10729 on WormBase ParaSite (Bolt et al., 2018)), *Meriones unguiculatus* (38,763 entries (Cheng et al., 2019)), *P. kellicotti* (12,850 entries, PRJNA179523 from NCBI (Schoch et al., 2020)), *L. loa* (12,473 entries, PRJNA246086 on WormBase ParaSite (Bolt et al., 2018)), *O. volvulus* (12,224 entries, PRJEB513 on WormBase ParaSite (Bolt et al., 2018)), *W. bancrofti* (13,525 entries, locally improved annotation of GCA_005281725.1 on GenBank (Sayers et al., 2023)) and *Felis catus* (40,213 entries, Uniprot (UniProt, 2023) download August, 2022). Searches were performed assuming the digestion enzyme was trypsin with a maximum of 4 missed cleavages allowed. For timsTOF a fragment ion mass tolerance of 50 ppm and a parent ion tolerance of 25 ppm were allowed, while for Q-Exactive data, a fragment ion mass tolerance of 20 ppm and a parent ion tolerance of 20 ppm were allowed. For data from all instruments, carbamidomethylation of cysteine was specified in Mascot as a fixed modification. Deamidation of asparagine, deamidation of glutamine, pyro-glutamate formation from n-terminal glutamine, acetylation of protein N-terminus and oxidation of methionine were specified as variable modifications. The resulting peptide and protein identifications were imported into Scaffold (Proteome Software, Inc., Portland, OR, USA; v 4.11.1) for visualization and post-processing filtering as described below.

3.7.2 Spectral Counts Analysis: All peptides that were assigned to *B.malayi* and *L. loa* protein databases were searched against protein databases from *M. unguiculatus* and *H. sapiens* using BLASTP, in order to discard any peptides which exactly matched host peptide sequences. After this processing, all peptides and associated spectral counts were parasite-specific sequences.

These processed spectral counts and peptide counts for all samples and all detected proteins are provided in **Supplementary Tables 4-6.** Predicted signal peptides were identified using the SignalP 6.0 server (Teufel et al., 2022) (excluding any proteins with ≥ 2 transmembrane domains, as identified by SignalP). Pathway enrichment was performed using WebGestalt v2019 (Liao et al., 2019) for KEGG pathways (Kanehisa et al., 2021) (Kyoto Encyclopedia of Genes and Genomes) and InterPro domains (Blum et al., 2021) while GOSTATS v2.50 was used for Gene ontology (GO) "molecular function" child term enrichment (Falcon and Gentleman, 2007). InterPro and GO annotations were performed using InterProScan v5.59-91.0 (Jones et al., 2014) and BlastKOALA v2.3 (Kanehisa et al., 2016) was used to annotate KEGG pathways. Relative protein abundance was quantified using Normalized Spectral Abundance Factor (NSAF) values, which are calculated per protein in each sample as the number of spectral counts (SpC) identified for that protein, divided by the protein's length in amino acids (L), divided by the sum of SpC/L of all protein in the experiment.

References

Ash, L.R., and Riley, J.M. (1970). Development of subperiodic Brugia malayi in the jird, Meriones unguiculatus, with notes on infections in other rodents. *J Parasitol* 56(5)**,** 969-973. doi: https://doi.org/10.2307/3277515.

Blum, M., Chang, H.Y., Chuguransky, S., Grego, T., Kandasaamy, S., Mitchell, A., et al. (2021). The InterPro protein families and domains database: 20 years on. *Nucleic Acids Res* 49(D1)**,** D344-D354. doi: 10.1093/nar/gkaa977.

Bolt, B.J., Rodgers, F.H., Shafie, M., Kersey, P.J., Berriman, M., and Howe, K.L. (2018). Using WormBase ParaSite: An Integrated Platform for Exploring Helminth Genomic Data. *Methods Mol Biol* 1757**,** 471-491. doi: 10.1007/978-1-4939-7737-6_15.

Chen, Z.W., Fuchs, K., Sieghart, W., Townsend, R.R., and Evers, A.S. (2012). Deep amino acid sequencing of native brain GABAA receptors using high-resolution mass spectrometry. *Mol Cell Proteomics* 11(1)**,** M111 011445. doi: 10.1074/mcp.M111.011445.

Cheng, S., Fu, Y., Zhang, Y., Xian, W., Wang, H., Grothe, B., et al. (2019). Enhancement of de novo sequencing, assembly and annotation of the Mongolian gerbil genome with transcriptome sequencing and assembly from several different tissues. *BMC Genomics* 20(1)**,** 903. doi: 10.1186/s12864-019-6276-y.

Contrepois, K., Ezan, E., Mann, C., and Fenaille, F. (2010). Ultra-high performance liquid chromatography-mass spectrometry for the fast profiling of histone post-translational modifications. *J Proteome Res* 9(10)**,** 5501-5509. doi: 10.1021/pr100497a.

Falcon, S., and Gentleman, R. (2007). Using GOstats to test gene lists for GO term association. *Bioinformatics* 23(2)**,** 257-258. doi: 10.1093/bioinformatics/btl567.

Hewitson, J.P., Harcus, Y.M., Curwen, R.S., Dowle, A.A., Atmadja, A.K., Ashton, P.D., et al. (2008). The secretome of the filarial parasite, Brugia malayi: proteomic profile of adult excretory-secretory products. *Mol Biochem Parasitol* 160(1)**,** 8-21. doi: 10.1016/j.molbiopara.2008.02.007.

Jones, P., Binns, D., Chang, H.Y., Fraser, M., Li, W., McAnulla, C., et al. (2014). InterProScan 5: genome-scale protein function classification. *Bioinformatics* 30(9)**,** 1236-1240. doi: 10.1093/bioinformatics/btu031

Kanehisa, M., Furumichi, M., Sato, Y., Ishiguro-Watanabe, M., and Tanabe, M. (2021). KEGG: integrating viruses and cellular organisms. *Nucleic Acids Res* 49(D1)**,** D545-D551. doi: 10.1093/nar/gkaa970.

Kanehisa, M., Sato, Y., and Morishima, K. (2016). BlastKOALA and GhostKOALA: KEGG Tools for Functional Characterization of Genome and Metagenome Sequences. *J Mol Biol* 428(4)**,** 726-731. doi: 10.1016/j.jmb.2015.11.006.

Liao, Y., Wang, J., Jaehnig, E.J., Shi, Z., and Zhang, B. (2019). WebGestalt 2019: gene set analysis toolkit with revamped UIs and APIs. *Nucleic Acids Res* 47(W1)**,** W199-W205. doi: 10.1093/nar/gkz401.

Loghry, H.J., Yuan, W., Zamanian, M., Wheeler, N.J., Day, T.A., and Kimber, M.J. (2020). Ivermectin inhibits extracellular vesicle secretion from parasitic nematodes. *J Extracell Vesicles* 10(2)**,** e12036. doi: 10.1002/jev2.12036.

Meier, F., Brunner, A.D., Koch, S., Koch, H., Lubeck, M., Krause, M., et al. (2018). Online Parallel Accumulation-Serial Fragmentation (PASEF) with a Novel Trapped Ion Mobility Mass Spectrometer. *Mol Cell Proteomics* 17(12)**,** 2534-2545. doi: 10.1074/mcp.TIR118.000900.

Mertins, P., Tang, L.C., Krug, K., Clark, D.J., Gritsenko, M.A., Chen, L., et al. (2018). Reproducible workflow for multiplexed deep-scale proteome and phosphoproteome analysis of tumor tissues by liquid chromatography-mass spectrometry. *Nat Protoc* 13(7)**,** 1632-1661. doi: 10.1038/s41596-018-0006-9.

Michalski, M.L., Griffiths, K.G., Williams, S.A., Kaplan, R.M., and Moorhead, A.R. (2011). The NIH-NIAID Filariasis Research Reagent Resource Center. *PLoS Negl Trop Dis* 5(11)**,** e1261. doi: 10.1371/journal.pntd.0001261.

Moreno, Y., and Geary, T.G. (2008). Stage- and gender-specific proteomic analysis of Brugia malayi excretory-secretory products. *PLoS Negl Trop Dis* 2(10)**,** e326. doi: 10.1371/journal.pntd.0000326.

Perkins, D.N., Pappin, D.J., Creasy, D.M., and Cottrell, J.S. (1999). Probability-based protein identification by searching sequence databases using mass spectrometry data. *Electrophoresis* 20(18)**,** 3551-3567. doi: 10.1002/(SICI)1522-2683(19991201)20:18<3551::AID-ELPS3551>3.0.CO;2-2.

Sayers, E.W., Cavanaugh, M., Clark, K., Pruitt, K.D., Sherry, S.T., Yankie, L., et al. (2023). GenBank 2023 update. *Nucleic Acids Res* 51(D1)**,** D141-D144. doi: 10.1093/nar/gkac1012.

Schoch, C.L., Ciufo, S., Domrachev, M., Hotton, C.L., Kannan, S., Khovanskaya, R., et al. (2020). NCBI Taxonomy: a comprehensive update on curation, resources and tools. *Database (Oxford)* 2020. doi: 10.1093/database/baaa062.

Teufel, F., Almagro Armenteros, J.J., Johansen, A.R., Gislason, M.H., Pihl, S.I., Tsirigos, K.D., et al. (2022). SignalP 6.0 predicts all five types of signal peptides using protein language models. *Nat Biotechnol* 40(7)**,** 1023-1025. doi: 10.1038/s41587-021-01156-3.

UniProt, C. (2023). UniProt: the Universal Protein Knowledgebase in 2023. *Nucleic Acids Res* 51(D1)**,** D523-D531. doi: 10.1093/nar/gkac1052.

Wisniewski, J.R., Zougman, A., Nagaraj, N., and Mann, M. (2009). Universal sample preparation method for proteome analysis. *Nat Methods* 6(5)**,** 359-362. doi: 10.1038/nmeth.1322.
